# Effects of Resveratrol and SIRT1 on PGC-1α Activity and Mitochondrial Biogenesis: A Reevaluation

**DOI:** 10.1371/journal.pbio.1001603

**Published:** 2013-07-09

**Authors:** Kazuhiko Higashida, Sang Hyun Kim, Su Ryun Jung, Meiko Asaka, John O. Holloszy, Dong-Ho Han

**Affiliations:** Division of Geriatrics and Nutritional Sciences, Department of Medicine, Washington University School of Medicine in St. Louis, Missouri, United States of America; University of Cambridge, United Kingdom

## Abstract

It has been reported that feeding mice resveratrol activates AMPK and SIRT1 in skeletal muscle leading to deacetylation and activation of PGC-1α, increased mitochondrial biogenesis, and improved running endurance. This study was done to further evaluate the effects of resveratrol, SIRT1, and PGC-1α deacetylation on mitochondrial biogenesis in muscle. Feeding rats or mice a diet containing 4 g resveratrol/kg diet had no effect on mitochondrial protein levels in muscle. High concentrations of resveratrol lowered ATP concentration and activated AMPK in C_2_C_12_ myotubes, resulting in an increase in mitochondrial proteins. Knockdown of SIRT1, or suppression of SIRT1 activity with a dominant-negative (DN) SIRT1 construct, increased PGC-1α acetylation, PGC-1α coactivator activity, and mitochondrial proteins in C_2_C_12_ cells. Expression of a DN SIRT1 in rat triceps muscle also induced an increase in mitochondrial proteins. Overexpression of SIRT1 decreased PGC-1α acetylation, PGC-1α coactivator activity, and mitochondrial proteins in C_2_C_12_ myotubes. Overexpression of SIRT1 also resulted in a decrease in mitochondrial proteins in rat triceps muscle. We conclude that, contrary to some previous reports, the mechanism by which SIRT1 regulates mitochondrial biogenesis is by inhibiting PGC-1α coactivator activity, resulting in a decrease in mitochondria. We also conclude that feeding rodents resveratrol has no effect on mitochondrial biogenesis in muscle.

## Abstract 2

Feeding resveratrol to rodents has no effect on mitochondrial biogenesis, and deacetylation of PGC-1α results in a decrease, not an increase, in its coactivator activity.

## Author Summary

Studies on cultured muscle cells have shown that treatment with resveratrol, a chemical famously found in the skin of red grapes, stimulates the manufacture of new mitochondria. This has been attributed to the activation of the deacetylase SIRT1 either directly by resveratrol or indirectly via the activation of AMP-activated protein kinase (AMPK). SIRT1 is then thought to deacetylate and activate the transcriptional coactivator PGC-1α, which in turn stimulates mitochondrial biogenesis. It has also been reported that feeding resveratrol to mice increases muscle mitochondria and results in improved running endurance. Here we further analyze the adaptive response of muscle mitochondria to resveratrol treatment to see if it mimics the response to endurance exercise. We find that feeding rats or mice large amounts of resveratrol did not increase muscle mitochondria. In these rodents, the bioavailability of oral resveratrol is low, and the resulting plasma level of resveratrol is far below the concentration required to activate AMPK. Contrary to previous reports we find that deacetylation by SIRT1 decreases PGC-1α activity and results in a decrease in mitochondria; moreover we show that the increase in mitochondria induced in cultured muscle cells by a high resveratrol concentration is due to the toxic activation of AMPK and, in turn, PGC-1α. However, this effect requires resveratrol concentrations that are very much higher than those attained by oral administration, and we conclude that oral resveratrol has no effect on mitochondrial biogenesis in skeletal muscle.

## Introduction

Resveratrol has been reported to have a number of remarkable effects in mice. These include protection against high-fat-diet-induced obesity and insulin resistance [1]–[3], marked improvements in running endurance and maximal oxygen uptake capacity (VO_2_max) [3], increased muscle strength [3], improved motor coordination [1]–[3], and antiaging effects [1],[2]. Subsequent studies have shown that resveratrol does not have antiaging effects in mice, as evidenced by no increases in average or maximum longevity [4],[5]. The protection against obesity and insulin resistance was attributed to an increase in, and uncoupling of, mitochondria in brown fat, and the increase in running endurance and VO_2_max were attributed to an increase in muscle mitochondria [3]. The increase in mitochondria induced by resveratrol was explained by activation of the protein deacetylase SIRT1, resulting in deacetylation and activation of the transcription coactivator PGC-1α [3]. PGC-1α regulates mitochondrial biogenesis [6]. The pharmaceutical agent SRT1720 has also been reported to activate SIRT1, resulting in PGC-1α activation, and an increase in enzymes of the mitochondrial fatty acid oxidation pathway in muscle and improved running performance, muscle strength, and coordination [7]. However, Pacholec et al. [8] have reported that SRT1720 does not activate SIRT1, and that it does not induce an increase in mitochondrial enzymes in mice. Based on studies on yeast and in vitro, it was initially thought that resveratrol directly activates SIRT1 [9]. However, Kaeberlein et al. [10] showed that, although resveratrol binds and deacetylates peptide substrates that contain a Fluor de Lys, it does not bind or deacetylate acetylated peptides lacking the flurophore. They also found that resveratrol has no effect on SIRT2 activity in yeast. Similarly, Bora et al. [11] found that resveratrol activation of SIRT1 was completely dependent on the presence of a covalently attached flurophore. Evidence that resveratrol can activate AMP activated protein kinase (AMPK) [12]–[14] led to further studies that indicated that the activation of SIRT1 by resveratrol is indirect, and is mediated by activation of AMPK [15]. The mechanism by which AMPK is thought to activate SIRT1 is by increasing NAD concentration [15].

We have a long-standing interest in the adaptive responses to endurance exercise, such as running and swimming, which include an increase in muscle mitochondria [16],[17]. Endurance exercise training also results in increases in endurance and in maximal oxygen uptake capacity. Endurance exercise does not, by itself, result in increases in either muscle strength, which occurs in response to heavy resistance exercise, or improved motor coordination, which occurs in response to activities that require various motor skills. A sedentary lifestyle greatly increases the risk of developing obesity, insulin resistance, type 2 diabetes, atherosclerosis, and frailty [18]. Therefore, in addition to being necessary for successful competition in sports, regular exercise is necessary for maintenance of health and functional capacity. Because it is difficult to motivate people to exercise, an effective, nontoxic exercise mimetic—that is, an “exercise pill”—could have great public health value. Therefore, the reports that, in addition to protecting against obesity and insulin resistance, resveratrol feeding mimics not only the adaptive response to endurance exercise but also the adaptations to strength training and motor skill exercise training were of great interest to us. The present study was undertaken to further evaluate the adaptive response of skeletal muscle mitochondria to resveratrol treatment.

## Results

### Studies on Rats and Mice

**Figure 1:**
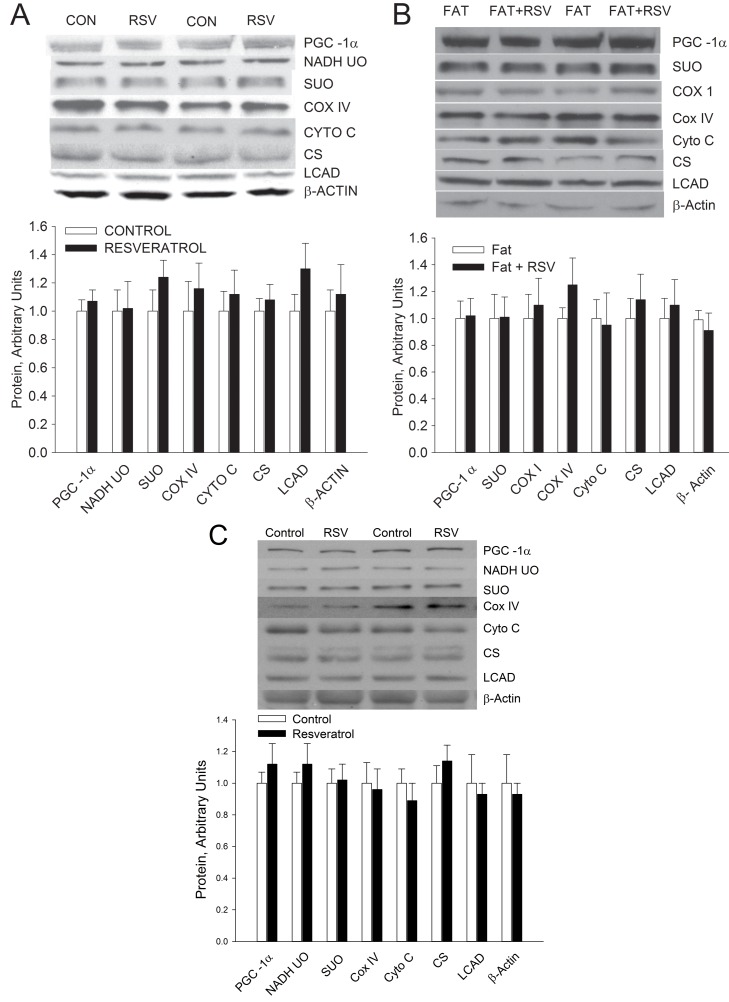
Feeding rodents resveratrol does not induce mitochondrial biogenesis in skeletal muscle. (A) Feeding rats a chow diet containing 4 g resveratrol (RSV) per kg diet for 8 wk had no effect on expression of PGC-1α or mitochondrial enzyme proteins in soleus muscle. (B) Feeding rats a high fat diet containing 4 g RSV per kg diet for 8 wk had no effect on expression of PGC-1α or mitochondrial enzyme proteins in triceps muscle. (C) Feeding mice a high fat diet containing 4 g resveratrol per kg diet had no effect on expression of PGC-1α or mitochondrial proteins in triceps muscle. Values are means ± SE for 6–8 muscles per group.

Feeding rats resveratrol in a chow diet containing 4 g resveratrol per kg diet [3] for 8 wk had no effect on the expression of PGC-1α or on a number of mitochondrial proteins in rat skeletal muscle as shown in soleus muscle (Figure 1A). A similar lack of effect was found in the gastrocenemius muscle. Feeding rats a high fat diet containing 4 g resveratrol per kg diet also had no effect on the expression of a range of mitochondrial enzyme proteins (Figure 1B). To rule out the possibility that the lack of effect of resveratrol on the mitochondrial content of skeletal muscle in rats was due to a species difference, we fed mice a high fat diet containing 4 g resveratrol per kg/diet as in the study by Lagouge et al. [3]. As in the rats, resveratrol feeding had no effect on the expression of PGC-1α or a number of mitochondrial proteins in skeletal muscle of mice (Figure 1C). To evaluate the possibility that the lack of effect of resveratrol on mitochondrial biogenesis is due to an inadequate increase in plasma resveratrol, we measured plasma resveratrol concentration. Plasma resveratrol concentration at 9:00 am in rats in the fed state averaged 1.56±0.28 µM. This plasma resveratrol concentration is higher than that reported by Lagouge et al. [3] in their resveratrol fed mice, in which the highest concentration attained was ∼0.5 µM.

### Studies on C_2_C_12_ Myotubes

**Figure 2:**
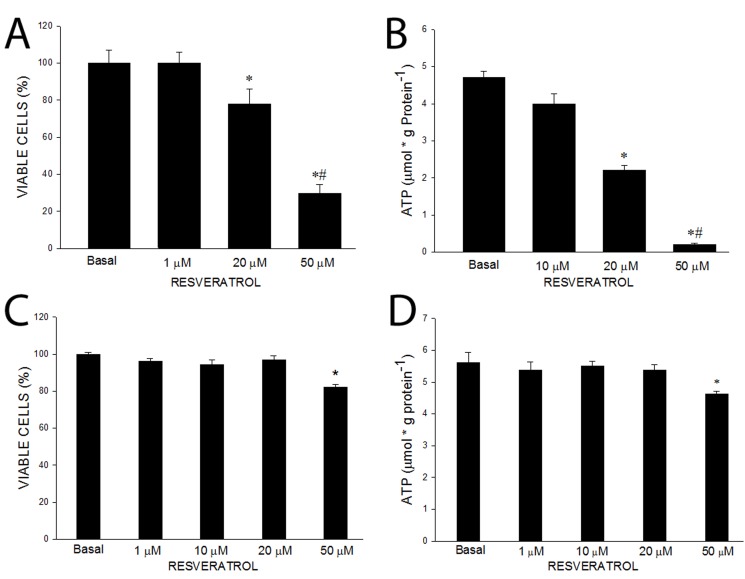
50 µM resveratrol is toxic to C_2_C_12_ myotubes. (A) Exposure to 50 µM resveratrol (RSV) for 24 h is toxic to C_2_C_12_ myotubes resulting in a decrease in viable cells and (B) a decrease in ATP content. Values are means ± SE for 4–6 experiments. (C) PGC-1α overexpression in C_2_C_12_ myotubes protects against the toxic effect of 24 h exposure to 50 µM RSV on viability and (D) ATP content. Values are means ± SE for six experiments. *p<0.05 versus basal.

Most of the information regarding the effects of resveratrol on, and the role of SIRT1 in, the regulation of mitochondrial biogenesis has come from studies on C_2_C_12_ myotubes or other cells in culture. Because resveratrol feeding had no effect on mitochondrial biogenesis in laboratory rodents, we evaluated the effect of resveratrol on mitochondrial biogenesis in C_2_C_12_ myotubes. The concentration of resveratrol that was routinely used in studies on C_2_C_12_ myotubes by Auwerx's group was 50 µM [3],[15], ∼100-fold higher than the highest plasma resveratrol level in their resveratrol fed mice [3]. In our initial experiments we found that 50 µM resveratrol is toxic, with a high proportion of the C_2_C_12_ myotubes appearing to be dead or dying after 24 h of exposure to 50 µM resveratrol. That this concentration of resveratrol is cytotoxic was born out by measurements of cytotoxicity (Figure 2A) and of ATP concentration, which was markedly reduced (Figure 2B). Similarly, Zang et al. [13] have reported that exposure of Hep-G2 cells to 50 µM resveratrol for 60 min resulted in an 80% reduction in ATP concentration. The decrease in ATP concentration in cells exposed to a high concentration of resveratrol is mediated by toxic effects on mitochondria, with inhibition of ATP synthase [19] and NADH: ubiquinone oxidoreductase [20]. Numerous studies have shown that concentrations of resveratrol in the 30 to 100 µM range kill a variety of malignant cells [21]. These studies were uncontrolled, and it was assumed that resveratrol specifically kills cancer cells. However, the present finding and that of Zang et al. [13] show that resveratrol at the high concentrations used is also lethal for nonmalignant cells.

### Effect of PGC-1α Overexpression

**Figure 3:**
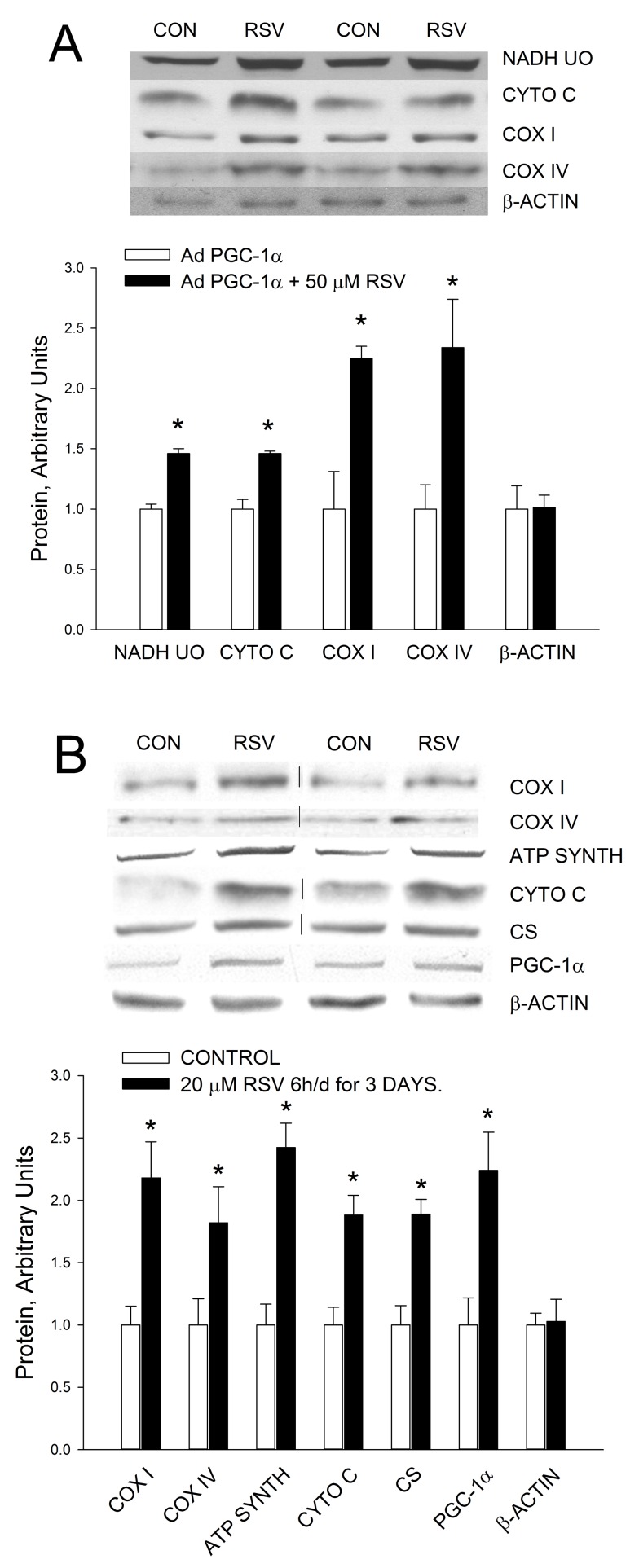
High concentrations of resveratrol induce an increase in mitochondrial proteins in C_2_C_12_ myotubes. (A) Treatment with 50 µM resveratrol (RSV) for 24 h resulted in an increase in mitochondrial proteins in C_2_C_12_ myotubes in which PGC-1α was overexpressed by infection with an adenovirus expressing PGC-1α (AdPGC-1α). Values are means ± SE for 7–8 experiments. (B) Treatment of wild-type C_2_C_12_ myotubes with 20 µM RSV for 6 h, followed by 18 h recovery, for 3 d induced increases in PGC-1α and mitochondrial proteins. Values are means ± SE for 6–8 experiments. *RSV versus control, *p*<0.05.

In the study in which 50 µM resveratrol increased mitochondrial biogenesis in C_2_C_12_ myotubes [3], the investigators used cells that overexpressed PGC-1α. We have observed that myotubes in which PGC-1α is overexpressed have increased resistance to the effect of puromycin (DH Han and JO Holloszy, unpublished findings), suggesting the possibility that overexpression of PGC-1α results in a nonspecific increase in resistance to toxins. We, therefore, evaluated the effect of 50 µM resveratrol in C_2_C_12_ cells in which PGC-1α was overexpressed by infection with a virus expressing PGC-1α. As shown in Figure 2C, the toxic effect of 24 h exposure to 50 µM resveratrol on cell viability was markedly reduced. However, there was still a significant reduction in ATP concentration (Figure 2D). Treatment with 50 µM resveratrol for 24 h resulted in an increase in mitochondrial biogenesis in the myotubes in which PGC-1α was overexpressed, as evidenced by increases in the expression of a number of mitochondrial proteins (Figure 3A). All of our subsequent experiments in which 50 µM resveratrol was used were performed on C_2_C_12_ myotubes in which PGC-1α was overexpressed.

### Effect of 20 µm Resveratrol in the Absence of PGC-1α Overexpression

To evaluate the effect of resveratrol in the absence of PGC-1α overexpression, we tried to identify a resveratrol concentration that induces an increase in mitochondrial proteins in wild type C_2_C_12_ cells. Resveratrol concentrations in the 1 µM to 10 µM range did not result in a decrease in ATP concentration (Figure 2B). Although exposure to 20 µM resveratrol for 24 h is less toxic than exposure to 50 µM, it results in a decrease in cell viability (Figure 2A) and a ∼50% decrease in ATP concentration (Figure 2B). Six hours of treatment with 20 µM resveratrol resulted in a smaller decrease in ATP (∼20%), and wild-type C_2_C_12_ cells treated with 20 µM resveratrol for 6 h followed by an 18 h recovery period showed no evidence of toxicity. “Training” the wild-type C_2_C_12_ cells by exposing them to 20 µM resveratrol for 6 h per day for 3 d resulted in increases in PGC-1α and a number of mitochondrial proteins (Figure 3B), while the same treatment with 1 µM, 5 µM, or 10 µM resveratrol had no effect (Figure S1).

### AMPK Is Necessary for the Resveratrol-Induced Increase in Mitochondrial Biogenesis

**Figure 4:**
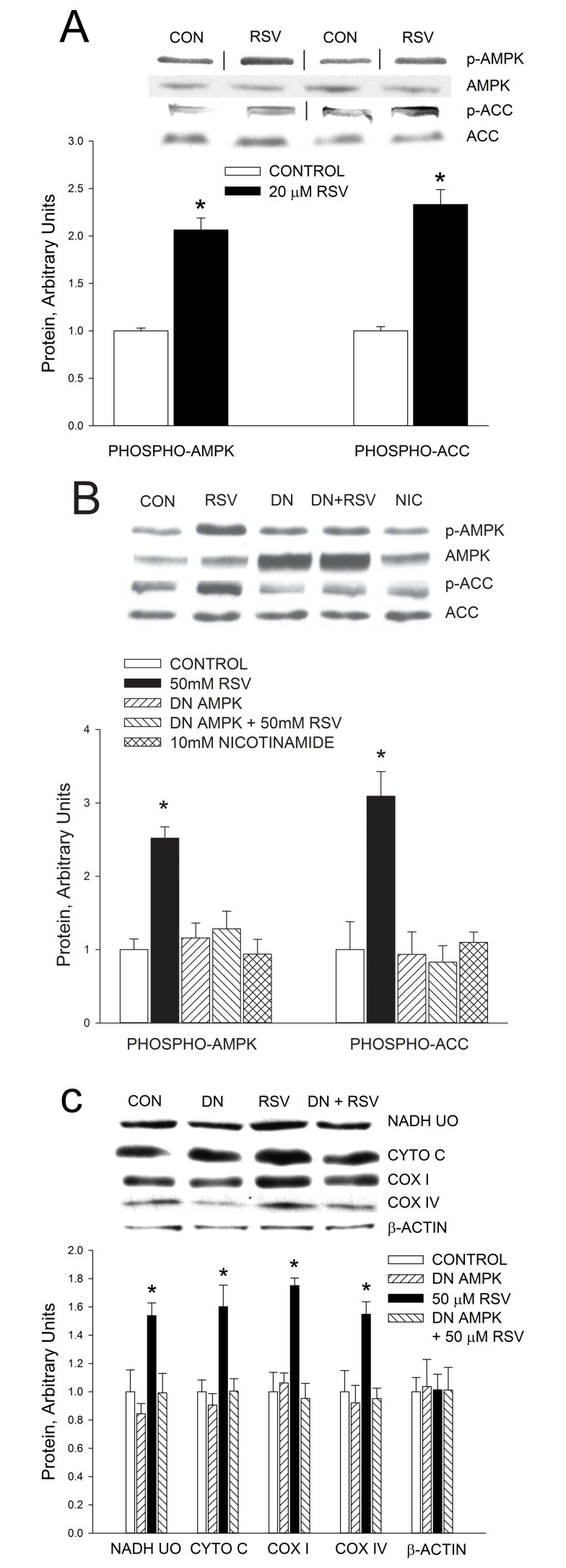
Resveratrol activates AMPK. (A) Treatment of C_2_C_12_ myotubes with 20 µM resveratrol (RSV) for 3 h results in increased phosphorylation of AMPK and acetyl CoA carboxylase (ACC). (B) Blocking AMPK activity by expression of dominant-negative AMPK (DN AMPK) in C_2_C_12_ myotubes prevents AMPK and ACC phosphorylation in response to RSV treatment. Nicotinamide had no effect on AMPK or ACC phosphorylation. (C) Blocking AMPK activity (DN AMPK) prevents induction of an increase in mitochondrial proteins by resveratrol. In experiments in which C_2_C_12_ myotubes were treated with 50 µM resveratrol, PGC-1α was overexpressed in the myotubes (see Figure 3 and text). Values are means ± SE for 6–8 experiments. *RSV versus other groups, *p*<0.05.

As shown in Figure 4A, treatment with 20 µM resveratrol resulted in increased phosphorylation of AMPK and acetyl-CoA carboxylase (ACC) in C_2_C_12_ myotubes. As a first step in evaluating the relative roles of AMPK and SIRT1 in the resveratrol-induced increase in mitochondrial biogenesis, we infected C_2_C_12_ myotubes with an adenovirus encoding a dominant-negative AMPK gene construct. That the DN AMPK was effective in blocking AMPK activity is demonstrated by prevention of increases in AMPK and ACC phosphorylation in response to resveratrol treatment (Figure 4B). Blocking AMPK activity prevented induction of an increase in mitochondrial proteins by resveratrol (Figure 4C), showing that AMPK activation is necessary for stimulation of mitochondrial biogenesis by resveratrol.

### Inhibition of SIRT1 Does Not Prevent the Resveratrol-Induced Increase in Mitochondrial Biogenesis

**Figure 5:**
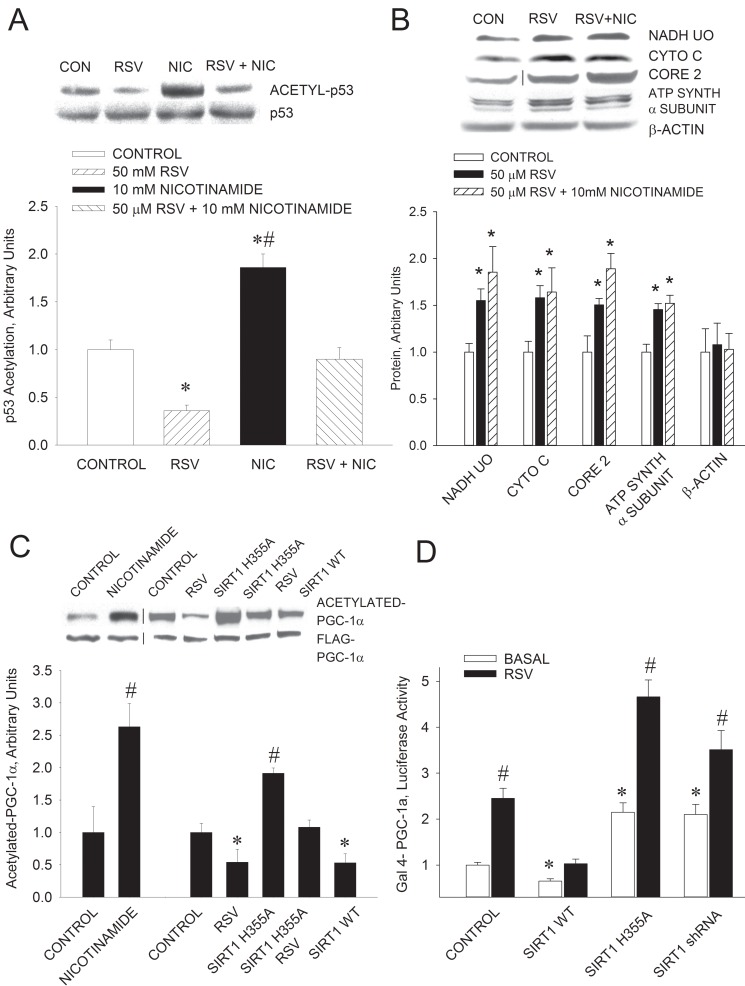
Suppression of SIRT1 activity increases PGC-1α acetylation and coactivator activity. (A) Nicotinamide (Nic) decreases SIRT1 activity in C_2_C_12_ myotubes as evidenced by increased p53 acetylation and prevention of p53 deacetylation in response to resveratrol (RSV). *p<0.05 versus control; ^#^ *p*<0.05 versus RSV and RSV+Nic. (B) Inhibition of SIRT1 with nicotinamide (Nic) does not prevent the resveratrol (RSV)-induced increase in mitochondrial proteins. *p<0.05 versus control. (C) Suppression of SIRT1 activity with nicotinamide or with dominant-negative SIRT1 H355A results in increased acetylation of PGC-1α. SIRT1 H355A reduces PGC-1α deacetylation in response to resveratrol (RSV) treatment, while overexpression of wild-type (WT) SIRT1 results in PGC-1α deacetylation. Values are means ± SE for 6–8 experiments. *p<0.05 versus control; ^#^ *p*<0.05 versus other groups. (D) PGC-1α coactivator activity, measured in C_2_C_12_ myotubes co-transfected with a PGC-1α GAL4 fusion product and a luciferase reporter, was increased by treatment with 20 µM resveratrol (RSV). Overexpression of wild-type (WT) SIRT1 resulted in reduced PGC-1α coactivator activity. Suppression of SIRT1 activity with dominant-negative SIRT1 H355A or knockdown of SIRT1 with SIRT1 shRNA resulted in increases in PGC-1α coactivator activity and potentiation of the effect of resveratrol on PGC-1α activity. In the experiments in which C_2_C_12_ myotubes were treated with 50 µM resveratrol, PGC-1α was overexpressed in the myotubes (see Figure 3 and text). Values are means ± SE for 6–7 experiments. *p<0.05 versus control; ^#^ *p*<0.05 versus basal.

Jäger et al. [22] have shown that AMPK directly phosphorylates and activates PGC-1α. Canto et al. [15] have interpreted their data to indicate that phosphorylation of PGC-1α by AMPK constitutes a priming event for subsequent deacetylation by SIRT1, and that deacetylation of PGC-1α is a key mechanism by which AMPK triggers PGC-1α activity. To further evaluate the relative roles of SIRT1 and AMPK in the resveratrol-induced increase in mitochondria, we used nicotinamide to inhibit SIRT1 [23]. That 10 mM nicotinamide decreases SIRT1 activity in C_2_C_12_ myotubes is evidenced by the finding of increases in the acetylation of p53, which is a SIRT1 substrate [24] (Figure 5A) and of PGC-1α (Figure 5C). Nicotinamide also prevented p53 deacetylation in response to 50 µM resveratrol (Figure 5A). However, we were surprised to find that nicotinamide did not prevent the resveratrol-induced increase in mitochondrial proteins (Figure 5B). Treatment of C_2_C_12_ myotubes with 10 mM nicotinamide had no effect on ATP concentration (nicotinamide 5.3±0.l3 µmol/g protein, Control 5.7±0.18; *n* = 6 per group).

### Effects of Suppressing or Increasing SIRT1 Activity

We further evaluated the role of SIRT1 in mitochondrial biogenesis by suppression of SIRT1 activity by adenovirus-mediated expression of a dominant-negative (DN) SIRT1 H355A [23], and knockdown of SIRT1 with a shRNA, in C_2_C_12_ myotubes. SIRT1 H355A suppressed SIRT1 activity as evidenced by an increase in PGC-1 acetylation and inhibition of resveratrol-induced PGC-1α deacetylation (Figure 5C). Both the DN SIRT1 and the SIRT1 shRNA resulted in increased PGC-1α coactivator activity, measured in C_2_C_12_ myotubes co-transfected with a PGC-1α–GAL4 fusion construct and a luciferase reporter [25], and enhanced the resveratrol-induced increase in PGC-1α activity (Figure 5D). Overexpression of wild-type SIRT1 resulted in PGC-1α deacetylation (Figure 5C), reduced PGC-1α coactivator activity, and prevented the increase in PGC-1α activity induced by resveratrol (Figure 5D).

**Figure 6:**
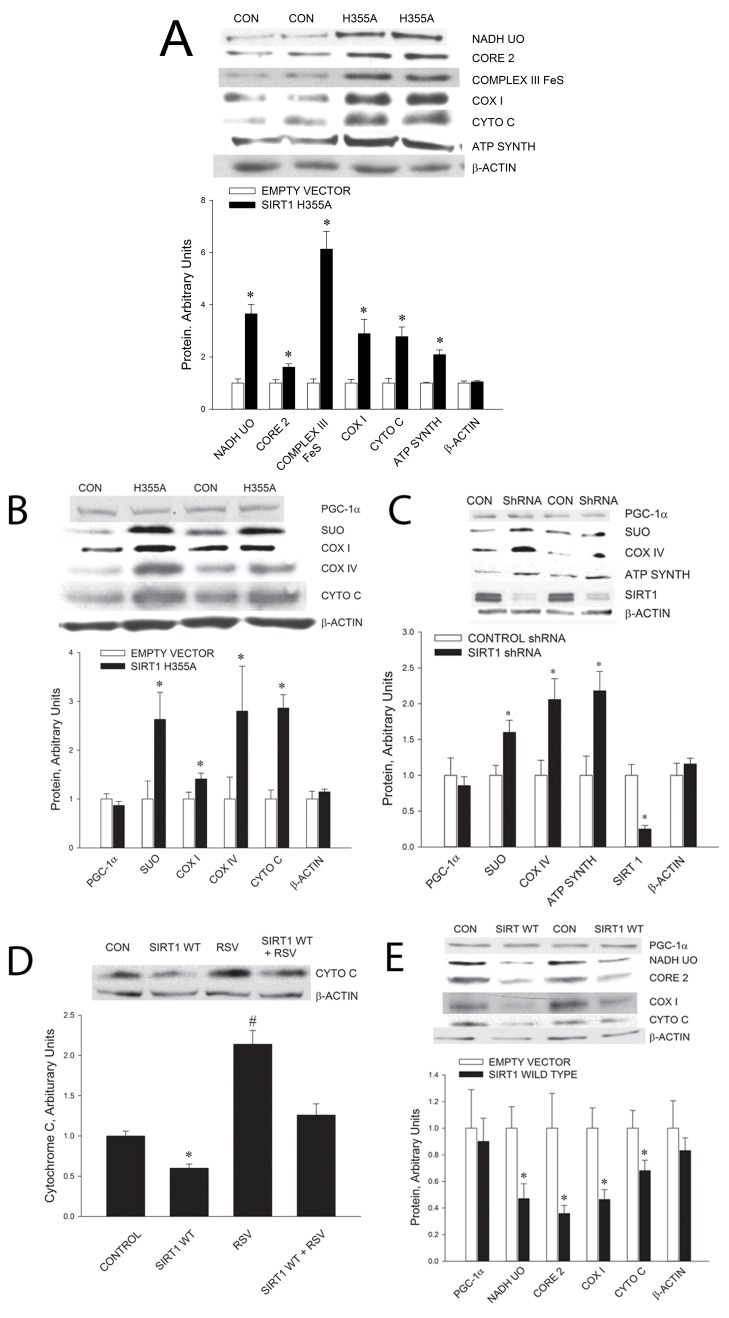
Reducing SIRT1 activity increases, and increasing SIRT1 expression decreases, mitochondrial enzymes in C_2_C_12_ myotubes and rat skeletal muscle. (A) Suppression of SIRT1 activity with dominant-negative SIRT1 H355A in C_2_C_12_ myotubes increases mitochondrial proteins. Values are means ± SE for 6–10 experiments per group. *p*<0.5 versus control. (B) Suppression of SIRT1 activity by expression of dominant-negative SIRT1 H355A in rat triceps muscle by electroporation resulted in increases in mitochondrial proteins. Values are means ± SE for 5–7 muscles per group. *p<0.05 versus control. (C) Knockdown of SIRT1 with a SIRT1 shRNA in C_2_C_12_ myotubes resulted in increases in mitochondrial proteins. Values are means ± SE for 6–8 experiments per group. *p<0.05 versus control. (D) Overexpression of wild-type (WT) SIRT1 in C_2_C_12_ myotubes resulted in decreased cytochrome c protein expression and inhibited the resveratrol (RSV)-induced increase in cytochrome c. Values are means ± SE for 6 experiments. *p<0.05 versus control. ^#^ *p*<0.05 versus other groups. (E) Overexpression of wild-type (WT) SIRT1 in rat triceps muscle by electroporation resulted in decreased expression of mitochondrial proteins. Values are means ± SE for 7–8 muscles per group. *p<0.05 versus empty vector.

SIRT1 H355A expression in myotubes resulted in an increase in mitochondrial enzyme proteins (Figure 6A). Expression of SIRT1 H355A in rat triceps muscle by electroporation also resulted in an increase in mitochondrial enzyme proteins (Figure 6B). Furthermore, knockdown of SIRT1 by transfection of C_2_C_12_ myotubes with a SIRT1 shRNA brought about an increase in mitochondrial proteins, providing further evidence that acetylation activates PGC-1α (Figure 6C). Expression of DN SIRT1 H355A in C_2_C_12_ myotubes had no effect on ATP concentrations (Control 5.7±0.18, DN SIRT1 H355A 6.0±0.3; *n* = 6 per group). To further evaluate the effect of SIRT1 on mitochondrial biogenesis, we determined the effect of overexpression of SIRT1 by adenovirus mediated infection of C_2_C_12_ cells, and electroporation of rat triceps muscle, with a SIRT1 gene construct. SIRT1 overexpression resulted in a decrease in cytochrome c and inhibited the resveratrol-induced increase in cytochrome c in C_2_C_12_ myotubes (Figure 6D). Overexpression of SIRT1 in rat triceps muscle resulted in decreases in mitochondrial enzyme proteins (Figure 6E).

Interestingly, the increase in PGC-1α coactivator activity induced by acetylation does not result in an increase in PGC-1α expression (Figure 6). This is in contrast to PGC-1α activation by phosphorylation by AMPK and/or p38 MAPK, which is associated with an increase in PGC-1α expression (Figure 3B) [22],[26]–[29]. A probable explanation for this difference is that AMPK and p38 MAPK do not just activate PGC-1, but also activate the transcription factors that induce increased PGC-1α expression. P38 MAPK phosphorylates and activates ATF2, which binds to a CREB binding site on the PGC-1α promoter, and AMPK and p38 MAPK bring about activation of MEF2, which binds to a MEF2 binding site on the PGC-1α promoter, resulting in increased PGC-1α transcription [27],[30]–[32].

## Discussion

In the present study, resveratrol feeding had no effect on mitochondrial biogenesis in skeletal muscle even though our animals were fed a diet containing the same amount of resveratrol, 4 g/kg diet, as used by Lagouge et al. [3], and more than the dose, 0.4 g/kg diet, used by Bauer et al. [1]. In studies on the effects of resveratrol on cells in culture, concentrations in the 30 µM to 100 µM range have routinely been used [3],[12],[14],[15]. Based on our findings on C_2_C_12_ myotubes, the concentration of resveratrol required to induce an increase in mitochondrial biogenesis is above 10 µM, and the data shown by Bauer et al. [1] suggest the concentration of resveratrol needed to activate AMPK in CHO cells is also above 10 µM. The plasma resveratrol concentration in our rats was 1.56±0.28 µM and the highest concentration in the mice of Lagouge et al. [3] was ∼0.5 µM. Thus, a likely explanation for the failure of resveratrol feeding to induce mitochondrial biogenesis in rats and mice in our study is its poor bioavailability.

In our experiments on C_2_C_12_ cells, we confirmed the finding of Lagouge et al. [3] that treatment of C_2_C_12_ cells with a high concentration of resveratrol results in both PGC-1α activation, evaluated using a PGC-1α–GAL4 construct together with a luciferase reporter, and an increase in mitochondrial biogenesis. The research groups of Auwerx and Puigserver have published a large number of studies, reporting that deacetylation activates and acetylation deactivates PGC-1α [3],[7],[15],[33]–[39]. Phosphorylation of PGC-1α by AMPK results in PGC-1 activation and increased mitochondrial biogenesis [22]. We found that high concentrations of resveratrol activate AMPK in C_2_C_12_ cells by a toxic effect on mitochondria that reduces ATP level, and that this is the mechanism by which resveratrol activates PGC-1α. We also found that the concomitant increase in SIRT1 activity, also mediated by AMPK, results in a deacetylation of PGC-1α that causes a blunting of the increase in PGC-1α activity induced by AMPK. This is in contrast to the report by Canto et al. [15] that activation of PGC-1α by AMPK is dependent on PGC-1α deacetylation by SIRT1. In support of this conclusion, they reported that inhibition of SIRT1 with nicotinamide or knock down of SIRT1 markedly reduced PGC-1α activation and attenuated the increase in mitochondrial proteins in response to AMPK activation.

We confirmed that activation of AMPK results in SIRT1 activation, as evidenced by deacetylation of p53 and PGC-1α. We also confirmed that suppression of AMPK activity blocks the increase in mitochondrial proteins induced by resveratrol. However, we were surprised to find that inhibiting SIRT1 with nicotinamide did not prevent the resveratrol-induced increase in mitochondrial proteins in C_2_C_12_ myotubes. Furthermore, an increase in PGC-1α acetylation, mediated by suppression of SIRT1 activity using a dominant-negative SIRT1 construct, resulted in an increase in PGC-1α coactivator activity and mitochondrial biogenesis. Knockdown of SIRT1 also increased PGC-1α activity. Further evidence that PGC-1 is activated by acetylation is provided by the findings that overexpression of wild-type SIRT1, resulting in PGC-1 deacetylation, decreases mitochondrial proteins, blunts the resveratrol/AMPK-induced increase in cytochrome c, and reduces PGC-1α coactivator activity. An additional mechanism by which the inhibitory effect of SIRT1 on PGC-1α activity might be mediated is by deacetylation and inactivation of the transacetylase p300 [40]. p300 is a transacetylase that binds to and acetylates PGC-1α [41], and powerfully enhances its coactivator activity [42]. Thus, inactivation of p300, resulting in decreased PGC-1α acetylation, could result in a reduction of PGC-1α activity.

Our findings that SIRT1 activation decreases PGC-1α coactivator activity and that inhibition or knockdown of SIRT1 increases PGC-1α activity are in keeping with data reported by Finkel's group [41]. These investigators showed that SIRT1 binds to and deacetylates PGC-1α, and that increasing SIRT1 expression in PC12 cells results in a ∼25% reduction in O_2_ consumption, a ∼45% decrease in cytochrome oxidase (COX) subunit 2 expression, and a ∼50% decrease in activity of a GAL4–PGC-1α fusion construct [41]. They also found that overexpression of the transacetylase p300, which activates PGC-1α [42], dramatically increased PGC-1α acetylation [41]. Our findings also confirm the report by Gurd et al. [43] that overexpression of SIRT1 in rat skeletal muscle results in decreased expression of the mitochondrial enzyme COXIV. Gurd et al. [43] also found an inverse relationship between mitochondrial content and SIRT1 content in different types of skeletal muscle and heart muscle.

SIRT1 is induced by, and appears to play a key role in the adaptive responses to, fasting, starvation, and calorie restriction [44]–[47]. The Auwerx and Puigserver research groups have interpreted their findings to indicate that SIRT1 leads to increased mitochondrial biogenesis, which provides a molecular mechanism that allows cells to survive and adapt to periods of nutrient deprivation [35], that SIRT1 activation by SRT1720 mimics low energy levels [7], and that “interdependent regulation of SIRT1 and AMPK provide a finely tuned amplifier mechanism for energy homeostasis under low energy availability” [15]. A key component of this concept is that mitochondrial adaptations induced by increased SIRT1 activity are necessary for the switch from carbohydrate to fat oxidation in response to fasting [7],[35]. What was actually reported is that treatment with resveratrol [3] or SIRT1720 [7] and other interventions that activated SIRT1 [34],[38] resulted in increases in basal oxygen consumption, heat production/body temperature, and protection against weight gain or reduced weight gain despite no decrease in food consumption. This syndrome, which resembles hyperthyroidism, was attributed by the authors to mitochondrial adaptations in brown adipose tissue and is incompatible with the large increase in running endurance reported in these mice [3],[7],[38].

Adaptive responses were selected for because they enhance an organism's chances of surviving environmental changes. Increases in energy expenditure and substrate oxidation resulting in more rapid depletion of energy stores, such as were reported to occur with SIRT1 activation, would be seriously maladaptive responses to fasting, starvation, or CR. Actually, it is well documented that fasting and CR result in decreases in metabolic rate, as reflected in lower resting oxygen consumption and a decrease in body temperature [48]–[51]. With regard to the claim that an increase in mitochondrial fatty acid oxidation enzymes is necessary for the switch from carbohydrate to fatty acid oxidation in muscle [7],[35], no increase in mitochondria is needed. Skeletal muscle has a low rate of energy utilization at rest and contains sufficient mitochondria to make possible a many-fold, acute increase in fatty acid oxidation in response to exercise that greatly exceeds the increase in fat oxidation in muscle in response to fasting. Furthermore, SIRT1-null mice are hypermetabolic, have elevated rates of fatty acid utilization, and readily switch from carbohydrate to fat oxidation in response to fasting [52].

In conclusion, our results show that resveratrol feeding does not induce an increase in muscle mitochondria in rodents. This lack of effect may be due to poor bioavailability, because the plasma levels of resveratrol attained in response to feeding large amounts of resveratrol are far below the concentration of resveratrol required to activate AMPK. This seems fortunate, because the activation of AMPK by resveratrol is mediated by a toxic effect that depletes ATP in cells exposed to AMPK-activating concentrations of resveratrol. Surprisingly, in light of the many studies reporting that deacetylation of PGC-1α results in activation of PGC-1α's coactivator activity, we find that deacetylation decreases, and PGC-1α acetylation increases, PGC-1α activity and mitochondrial biogenesis. Our results indicate that the activation of PGC-1α by resveratrol is mediated by AMPK, and that the activation of SIRT1 by AMPK acts to reduce, rather than induce, this activation.

## Experimental Procedures

### Ethics Statement

This research was approved by the Animal Studies Committee of Washington University School of Medicine. Rats were lightly anesthetized during muscle electroporation. Rats were anesthetized with pentobarbital and, after muscles were harvested, were killed by exsanguination while under anesthesia.

### Materials

Antibodies against cytochrome oxidase subunit I (COXI), cytochrome oxidase subunit IV (COX IV), Core II, Complex III FeS, NADH ubiquinol oxidoreductase (NADH-UO), and succinate ubiquinol oxidoreductase(SUO) ATP synthase alpha subunit #45924 and lipofectamine 2000 were purchased from Invitrogen (Carlsbad, CA). Anti-cytochrome *c* antibody was obtained from BD Biosciences (San Jose, CA). Antibodies against p53, acetyl-p53, AMP-activated protein kinase (AMPK), phospho-AMPK, acetyl-CoA carboxylase (ACC) and phospho-ACC were products of Cell Signaling technology (Beverly, MA). An anti-SIRT1 antibody #09844 was purchased from EMD Millipore. An anti-PGC-1α c-terminal (777–797) antibody #516557 was purchased from EMD Millipore (Billerica, MA); an antibody against acetylated lysine #9441 was purchased from Cell Signaling (Beverly, MA). Horseradish peroxidase (HRP)–conjugated donkey anti-rabbit IgG and donkey anti-mouse IgG were purchased from Jackson ImmunoResearch Laboratories (West Grove, PA). Enhanced chemiluminescence (ECL) reagents were obtained from Amersham (Arlington Heights, IL). All other reagents were purchased from Sigma (St. Louis, MO).

### Resveratrol Feeding Studies

This research was approved by the Animal Studies Committee of Washington University School of Medicine. Male Wistar rats weighing ∼95 g were purchased from Charles Rivers (Wilmington, MD) and housed in individuals cages. The resveratrol used in the study on rats was purchased from Stryka Botanicals (Hillsborough, NJ). Control rats were fed Purina rodent chow, and the resveratrol-fed rats were given the chow diet containing 4 g resveratrol per kg diet, for 8 wk. Male c57BL/6J mice were purchased from Jackson Laboratory (Bar Harbor, ME), housed 6 per cage, and fed a high fat diet, 50% of calories from fat, or the high fat diet containing 4 g resveratrol per kg diet 3 for 8 wk. The resveratrol used in the study on mice was a kind gift from DSM Nutritional Products (Basel, Switzerland). (The resveratrol used in the study by Lagouge et al. was from Orchid, Chennai, India.)

### Muscle Harvesting

Rats or mice were anesthetized with sodium pentobarbital 5 mg/100 g body weight. Muscles were dissected out, clamp-frozen, and kept at −80°C until used for assays.

### C_2_C_12_ Myotube Viability Evaluation

ATP concentration was measured using a luminescence ATP detection assay (ATPlite, Perkin Elmer, Waltham, MA); LDH activity, as an indicator of cytotoxicity, was measured using an LDH-Cytotoxicity Assay Kit (BioVision, Mountain View, CA), according to the manufacturer's instructions.

### Constructs

For expression in skeletal muscle via electroporation, wild-type SIRT1 and dominant-negative SIRT-1 H355A constructs were purchased from Addgene (Cambridge, MA) and inserted into pCDNA3.1 (Invitrogen, Carlsbad, CA). For expression in C_2_C_12_ myoblasts by transfection, a gal-4-DBD-PGC-1α plasmid was purchased from Addgene (Cambridge, MA) [53], a 9×gal-4–dependent reporter plasmid was purchased from Promega (Madison, WS), and a LacZ control plasmid was purchased from Invitrogen (Carlsbad, CA). For expression by adenoviral infection in C_2_C_12_ myotubes, the adenoviral constructs of pAd-Track Flag-PGC-1α [36], pAd-Track Flag-SIRT1 [23], and pAd-Track Flag dominant-negative SIRT1 H355A [23] were purchased from Addgene (Cambridge, MA). Dominant-negative Myc-AMPKα 2 DNA [54] was PCR cloned and ligated into pAd-Track plasmid. Mouse SIRT1 shRNA (5′-GCCCTGTAAAGCTTTCAGAA-3′) and scrambled control (5′-GATGAAGTCGACCTCCTCAT-3′) sequences were cloned into pRNAT-H1.1/adeno (Genescript, Piscataway, NJ). Recombinant adenoviruses were generated employing a system described by He et al. [55].

### Muscle Electroporation

Transfection of DNA into rat skeletal muscle was accomplished by using an electric pulse-mediated gene transfer technique [56]. Male Wistar rats weighing ∼60 g were anesthetized with isoflurane gas. A triceps muscle was injected with 100 µg of plasmid DNA containing either empty vector, pcDNA3.1 SIRT1 WT, or pcDNA3.1 Sirt1 H355A in 100 µl saline, using a 27 gauge needle, at a rate of 0.04 ml/min. After injection, an electric field was applied to the triceps muscle using a S88 square-pulse stimulator (Grass) with a 533 model two-needle array (BTX). The electric field application consisted of 8 pulses of 100 ms duration, at a frequency of 1 Hz and amplitude of 100 volts, that were applied perpendicular to the muscles' long axis. Muscles were harvested 14 d after electroporation.

### Cell Culture, Treatments, and Adenoviral Infections

C_2_C_12_ mouse myoblasts were grown in DMEM (4.5 g glucose/L, Invitrogen) containing 10% fetal bovine serum, 100 µU/ml penicillin, and 100 µU/ml streptomycin. Differentiation was initiated by switching to medium containing 2% heat inactivated horse serum when the myoblasts were 90% confluent. After 48 h of differentiation, batches of myotubes were infected with adenoviruses expressing (a) Flag-PGC-1α, (b) dominant-negative Myc-AMPKα 2, (c) dominant-negative Flag-SIRT1 H355A, (d) Flag-SIRT1, and (e) SIRT1 shRNA. At 96 h after differentiation, batches of C_2_C_12_ myobutes were treated with 20 µM or 50 µM resveratrol or vehicle for the time periods given in the figures, or with 10 mM nicotinamide or vehicle for 24 h.

### Western Blotting

Homogenates were prepared and Western blotting was performed as described previously [57] using the antibodies described previously [57],[58].

### PGC-1α Activity Assay

To evaluate the effect of SIRT1 on PGC-1α transcription coactivator activity, C_2_C_12_ myoblasts were co-transfected with a gal-4-DBD PGC-1α plasmid, and a 9×gal-4-dependent reporter plasmid, or with a LacZ control plasmid, and with either wild-type SIRT1, dominant-negative SIRT1 H355A, or SIRT1 shRNA-plasmids using lipofectamine 2000. After overnight transfection the culture medium was changed to DMEM containing 10% FBS. Thirty-six hours later, some of the cells were treated with 20 µM resveratrol for 6 h and harvested after a 6 h recovery period. Dual luciferase assays were performed using a kit (Invitrogen) according to the manufacturer's instructions.

### PGC-1α Acetylation

Flag-PGC-1α was expressed in C_2_C_12_ myotubes by adenoviral infection. To evaluate the effect of SIRT1 on PGC-1α acetylation, the myotubes were co-infected with wild-type SIRT1 or SIRT1 H355A. Forty-eight hours after infection, myotubes were treated with 50 µM resveratrol or vehicle for 18 h. Wild-type C_2_C_12_ myotubes were treated with 10 mM nicotinamide for 24 h. The myotubes were then harvested, and cell extracts containing 200 µg of protein were rotated with anti-Flag antibody at 4°C overnight. The following morning, agarose G beads were added and the samples were rotated at room temperature for 2 h. The agarose beads were washed 4 times with PBS and protein was eluted from the beads with 5× SDS buffer, which was boiled for 5 min. PGC-1α was measured with an anti-PGC-1α antibody, and levels of PGC-1α acetylation were then assessed with an anti-acetyl lysine antibody (#9441 Cell Signaling Technology).

### Statistical Analysis

Results are expressed as means ± SE. The significance of differences between two groups was determined using Student's *t* test. For multiple comparisons, significance was determined by one-way analysis of variance followed by post hoc comparison using Tukey significant difference method.

## Supporting Information

Figure S1 Treatment of C_2_C_12_ myotubes with 1.0 µM, 5 µM, or 10 µM of resveratrol for 24 h had no effect on expression of PGC-1α or mitochondrial enzyme proteins. (TIF)

## Abbreviations

ACC: acetyl-CoA carboxylase
AMPK: AMP-activated protein kinase
COX: cytochrome oxidase
DN: dominant-negative
ECL: enhanced chemiluminescence
HRP: Horseradish peroxidase
NADH-UO: NADH ubiquinol oxidoreductase
SUO: succinate ubiquinol oxidoreductase
VO_2_max: maximal oxygen uptake capacity
