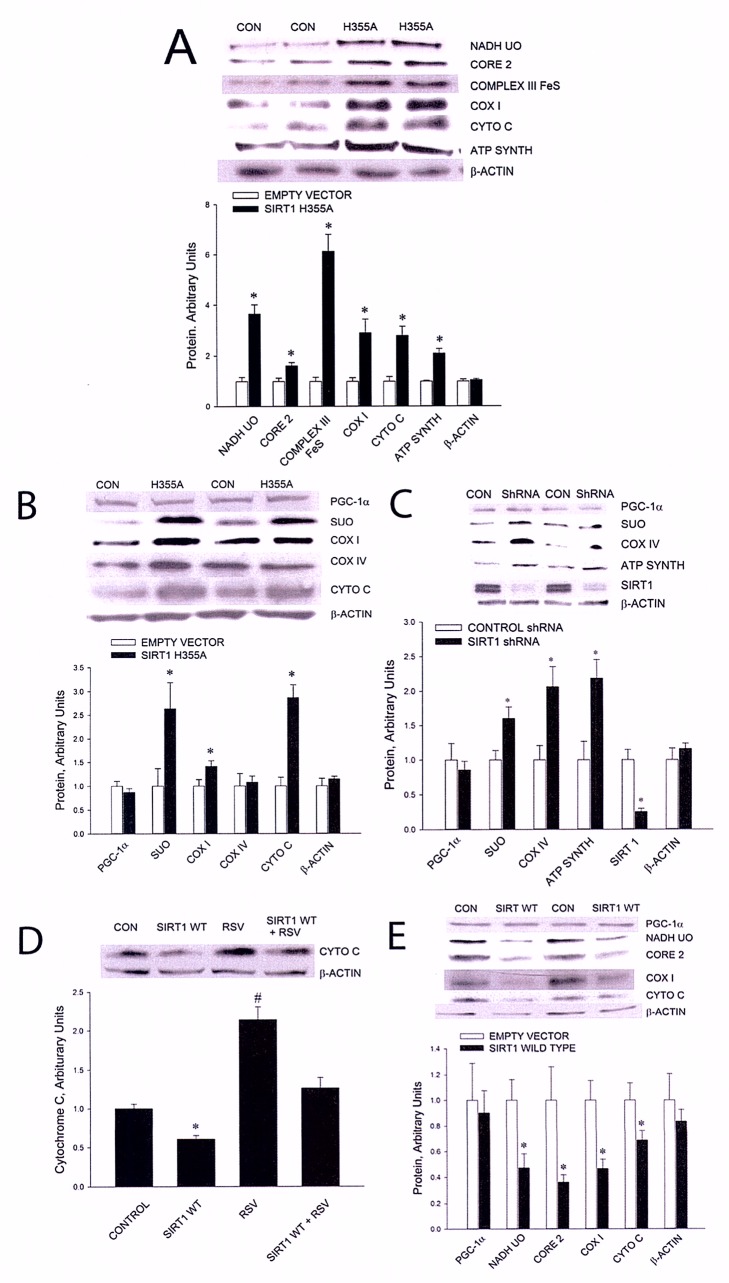# Correction: Effects of Resveratrol and SIRT1 on PGC-1α Activity and Mitochondrial Biogenesis: A Reevaluation

**DOI:** 10.1371/annotation/900c9397-eeb9-4d4e-9a05-ff18d657be79

**Published:** 2014-01-29

**Authors:** Kazuhiko Higashida, Sang Hyun Kim, Su Ryun Jung, Meiko Asaka, John O. Holloszy, Dong-Ho Han

The authors inadvertently used the wrong blots in three of the main figures resulting in duplications. The blots in question are PGC-1a and LCAD in Figure 1A, Cyto C in Figure 4C, and COXIV in Figure 6B. The authors and their institution have confirmed that these errors do not affect the interpretation of the results or conclusions of the paper. The authors apologize for any confusion caused by these mistakes and thank the readers for alerting them to these issues.

Please view the correct Figure 1 here:

**Figure:**
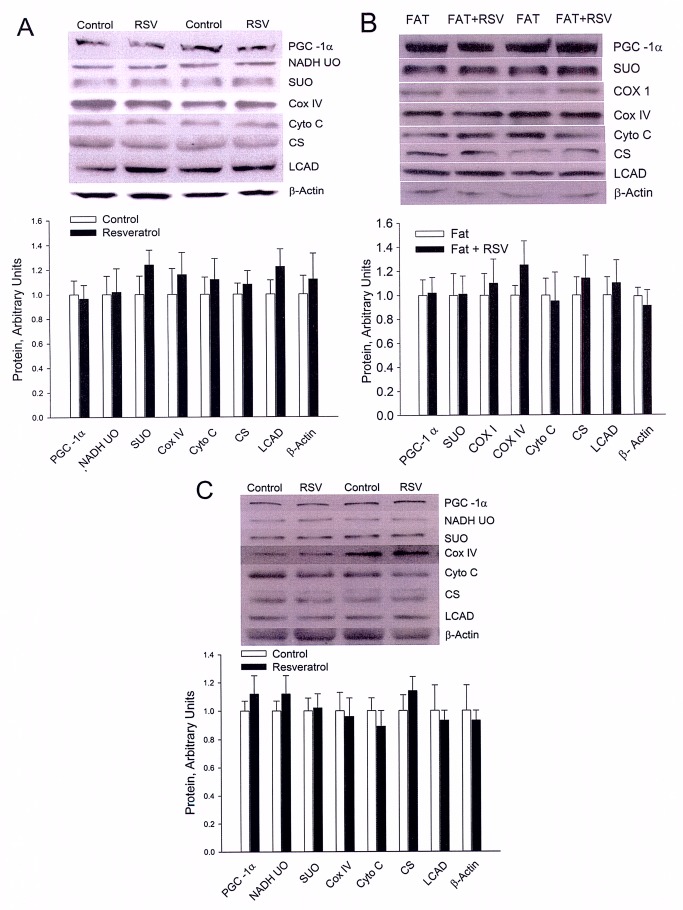


Please view the correct Figure 4 here:

**Figure:**
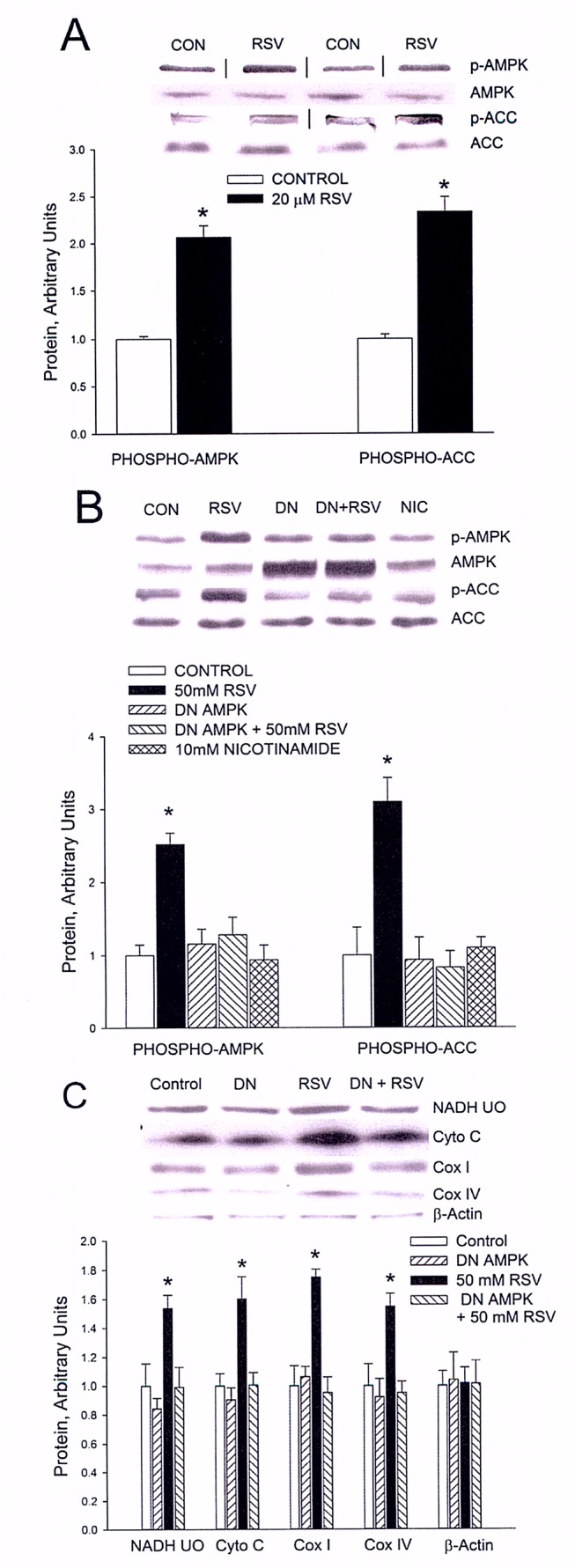


Please view the correct Figure 6 here:

**Figure:**