# Supplementary figures and images for: Effects of Resveratrol and SIRT1 on PGC-1α Activity and Mitochondrial Biogenesis: A Reevaluation
Source: PLoS Biol. 2013 Jul 9;11(7):e1001603. doi: 10.1371/journal.pbio.1001603 (PMC3706311; doi:10.1371/journal.pbio.1001603)

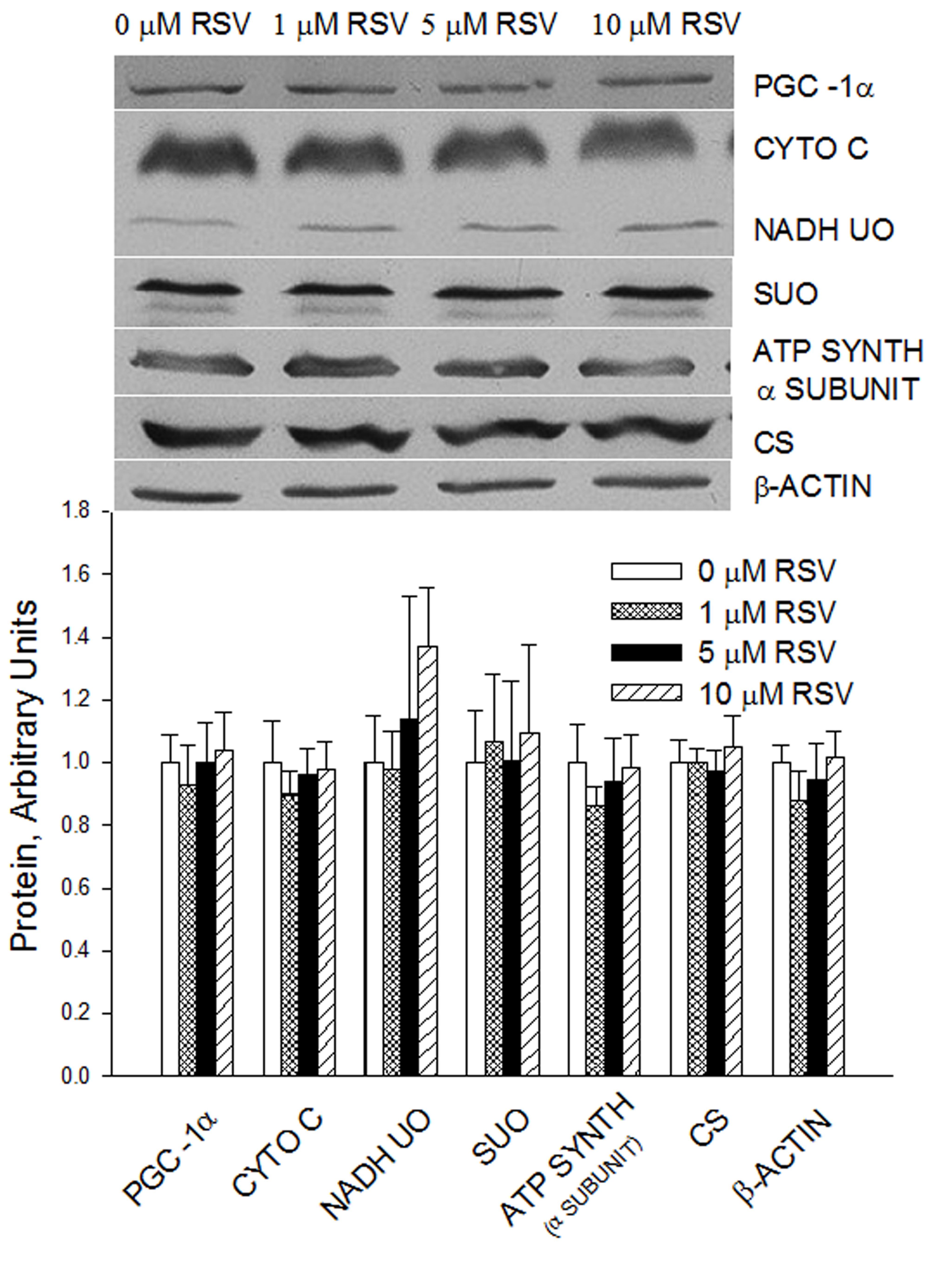

Supplement: Figure S1 — (TIF) [file pbio.1001603.s001.tif]
